# An Approach to Evaluation of the Effect of Bioremediation on Biological Activity of Environmental Contaminants: Dechlorination of Polychlorinated Biphenyls

**DOI:** 10.1289/ehp.6935

**Published:** 2004-12-09

**Authors:** Patricia E. Ganey, Steven A. Boyd

**Affiliations:** Departments of ^1^Pharmacology and Toxicology, and; ^2^Crop and Soil Science, Institute of Environmental Toxicology, Michigan State University, East Lansing, Michigan, USA

**Keywords:** bioassay, cytochrome P450, dechlorination, insulin, *in vitro* fertilization, neutrophil, PCB, transcription, uterine contraction

## Abstract

The effectiveness of bioremediation efforts is assessed traditionally from the loss of the chemical of interest. In some cases, analytical techniques are coupled with evaluation of toxicity to organisms representative of those found in the affected environment or surrogate organisms. Little is known, however, about the effect of remediation of environmental chemicals on potential toxicity to mammalian organisms. We discuss both an approach that employs mammalian cell system bioassays and the criteria for selection of the assays. This approach has been used to evaluate the biological response to mixtures of polychlorinated biphenyls (PCBs) before and after remediation by reductive dechlorination. The dechlorination process used results in accumulation of congeners substituted in only the *ortho* and *para* positions and containing fewer chlorines than the starting mixtures. Evaluation of the dechlorinated mixture reveals a loss of biological activity that could be ascribed to coplanar PCBs not containing chlorine in the *ortho* positions. Conversely, biological activity associated with *ortho*-substituted PCB congeners is unaffected or increased by remediation. Thus, the results of the bioassays are consistent with the remediation-induced change in the profile of PCB congeners and the known mechanisms of action of PCBs. The results emphasize a need for evaluation of the products of remediation for biological activity in mammalian systems. Furthermore, the approach outlined demonstrates the potential to assess the impact of remediation on a range of biological activities in mammalian cells and thus to estimate positive and negative effects of remediation strategies on toxicity. Future needs in this area of research include assays to evaluate biological effects under conditions of exposure that mimic those found in the environment and models to extrapolate effects to assess risk to people and wildlife.

Biological remediation technologies offer the advantage of partial or complete destruction of contaminants within a site. The ultimate goal of remediation is conversion of toxic organic contaminants to simple, less-toxic constituents, although for some chemicals, incomplete conversion occurs and stable intermediates are formed. The effectiveness of remediation strategies is traditionally evaluated from the disappearance of the chemical of interest. This approach does not consider that end products or intermediates produced during remediation may be toxic. Furthermore, the potential exists that remediation may result in products for which the toxic response is greater than for the parent compound or for which the target of toxicity is different, and these possibilities would not be detected. Accordingly, from the standpoint of assessing risk, it is important to understand the biological activity or toxicity of the end products and stable intermediates. Thus, the question becomes, Are the products or intermediates of bioremediation less toxic than the starting materials?

The anticipated answer to this question is yes; however, there is a dearth of evidence to support this assumption, particularly with respect to effects on mammalian systems. There are some reports of decreased toxic effects after remediation of contaminants, using mammalian systems to evaluate toxicity (Mousa et al. 1996, 1998; Quensen et al. 1998). On the other hand, some evidence suggests that products formed during remediation or breakdown of environmental chemicals have greater biological activity than the starting materials. For example, DDE [1,1-dichloro-2,2-bis(*p*-chlorophenyl)ethylene], a major environmental transformation product of DDT [1,1,1-trichloro-2,2-bis(*p*-chlorophenyl) ethane], is a more potent androgen receptor antagonist than its parent compound (Kelce et al. 1995). In addition, products of microbial reductive dechlorination of polychlorinated biphenyls (PCBs) are more effective than parent PCB mixtures at stimulating uterine contractions *in vitro* (Bae et al. 2001). Similarly, chemical remediation may result in products with increased biological activity. For example, pyrene, a four-ringed polycyclic aromatic hydrocarbon, can be degraded with ozone. This ozonation results in the formation of at least 10 major products, some of which are more mutagenic than pyrene itself (Sasaki et al. 1995). The initial products formed from ozonation of a variety of polycyclic aromatic hydrocarbons in aqueous solution cause greater inhibition of the ability of mammalian cells to communicate through gap junctions compared with the parent compounds (Upham et al. 1997; Weis et al. 1998). These reports emphasize the need for investigators to consider the biological activity not only of the parent contaminants, but also of their stable transformation products produced during remediation.

## Bioassays Commonly Used to Assess Effectiveness of Remediation

Investigators have not ignored the question of whether loss of biological activity accompanies remediation. The approaches used include bioassays using organisms representative of those we expect to find in the affected environment or surrogate organisms or plants. For example, the survival, growth, and reproduction of a variety of marine organisms exposed to sediments or soil collected from contaminated sites before and after remediation have been used to assess effectiveness of some remediation strategies [Deanovic et al. 1999; Kemble et al. 2000; McGann et al. 2003; Tabak et al. 2003; U.S. Environmental Protection Agency (EPA) 1989]. Toxicity to earthworms has been used to evaluate the effects of methods of removal of contaminants from soil (Chang et al. 1997; Maenpaa et al. 2002; Saterbak et al. 1999; U.S. EPA 1988). Luminescent bacterial assays such as the commercially available Microtox assay have also been used widely (Ahtiainen et al. 2002; Dorn and Salanitro 2000; Frische and Hoper 2003; Kemble et al. 2000; Layton et al. 1999). This technique is based on the observation that some bacteria (e.g., *Vibrio fischeri*) luminesce in proportion to cellular metabolism; accordingly, toxicity to the microorganisms is detected as a decrease in the intensity of luminescence. A solid-phase application of this method offers an advantage in that it permits exposure of bacteria to sediment-bound contaminants (Kemble et al. 2000). An integrated approach to ecotoxicologic evaluation involves combinations of these methods (Frische 2003).

These approaches yield valuable information regarding effectiveness of remediation and help focus additional remediation strategies. As with all bioassays, each has advantages and disadvantages, some of which relate to sensitivity, cost, versatility of application, reliability, rapidity, reproducibility, and relationship to health risk. A comprehensive discussion of these is not within the scope of this work. However, none of these bioassays addresses the potential biological activity of products of remediation in mammalian systems that may represent more specific and/or integrated functions relevant to human health. In the remainder of this article, we review an approach to the evaluation of toxicity of products of remediation in mammalian systems.

## Bioassays Employing Mammalian Cell Systems

The concept that products of remediation may have biological activity in mammalian systems has not been studied extensively. Investigators associated with the Michigan State University Superfund Program Project began an effort a number of years ago as part of a Bioremediation Product Evaluation Core to address the issue. The working hypothesis was that products of remediation have different biological activities compared with those of the starting compounds or mixtures. We developed a list of assays of biological activity that relied on the strengths and expertise of the toxicologists within the group (Table 1). Generally, criteria for useful bioassays include sensitivity over a range of concentrations of test chemical, low rate of false-positive and false-negative responses, ease and rapidity of the assay, reproducibility of results, and reasonable cost. How well the end point being measured reflects a biological response of interest in humans or animals may also be important. For purposes of using results from an assay for risk assessment, it is helpful to have a reference value for toxicity, namely, a response known to be associated with toxicity in whole organisms. Assays selected for use in the Bioremediation Product Evaluation Core met many of these criteria. Additional criteria for inclusion in the Core were that assays were performed routinely within a laboratory and that the expected results were relatively uncomplicated in interpretation. These latter two criteria precluded the use of whole-animal studies, so the assays selected involved *in vitro* methodology. With this approach, the list developed covers a variety of cellular functions including intracellular signaling, intercellular communication, proliferation and cell death, gene expression, measures of integrated cellular function and integrated tissue function, and aryl hydrocarbon (Ah) receptor function (important for dioxin-like contaminants) (Table 1). Accordingly, although the list is not exhaustive, many possible responses to chemical insult are represented. Additional measures not represented on this list that would be useful include whole-animal assessments and assays that measure endocrine disruption, neurotoxicity, genotoxicity, or mutagenicity.

In evaluating remediation products, we selected specific bioassays for initial examination on the basis of current knowledge of the mechanism of action of the parent compound of interest. For example, for dioxin-like chemicals (e.g., PCBs) one of the first avenues of investigation was the effects on cytochrome P450 induction based on the known activity of these compounds to increase cytochrome P4501A. Similarly, for chemicals known to disrupt intracellular signaling, such as some of the polycyclic aromatic hydrocarbons (Burdick et al. 2003; Patten Hitt et al. 2002), first priority for analysis was given to examination of activation of mitogen-activated protein kinases or alterations in neutrophil function. Initial studies using this approach were aimed at evaluation of products of bioremediation of PCBs. One promising remediation technique for PCBs is the removal of chlorines by microorganisms. We review results of these studies below.

## Evaluation of Products of Reductive Dechlorination of PCBs

Polychlorinated biphenyls are among the most widely distributed environmental contaminants. Commercial PCB mixtures were manufactured in the United States between 1929 and 1978 and used for a variety of industrial purposes. An estimated 1.4 billion pounds of PCBs have been produced worldwide and approximately several hundred million pounds have been released into the environment. Commercial PCBs (e.g., Aroclors) typically consist of 60–90 of the 209 possible congeners, each of which differs in the positions and/or numbers of chlorines on the biphenyl ring. Several characteristic PCB mixtures differ in the extent of chlorination and specific congener composition. Common examples are Aroclors 1242, 1248, and 1254, which contain 42, 48, and 54% chlorine by weight, respectively. Because of their lipophilic properties, PCBs tend to accumulate in biological tissue and in environments rich in organic matter, such as sediments.

PCB mixtures found in the environment often do not match any of the known commercial formulations because they have been subjected to congener-selective environmental processes, for example, reductive dechlorination by anaerobic bacteria (Bedard and Quensen 1995; Quensen et al. 1988, 1990). Reductive dechlorination is a microbially mediated process that removes chlorine from biphenyl with replacement by hydrogen, resulting in a product mixture in which the average number of chlorines is substantially diminished. Chlorines substituted in the *meta* and *para* positions are preferentially removed by this process; *ortho* chlorines are rarely removed. *In situ* reductive dechlorination has been documented in anaerobic sediments at numerous locations, and six distinct dechlorination patterns have been observed, giving rise to six recognizable profiles of congeners in the dechlorination products (Bedard and Quensen 1995).

**Figure 1:**
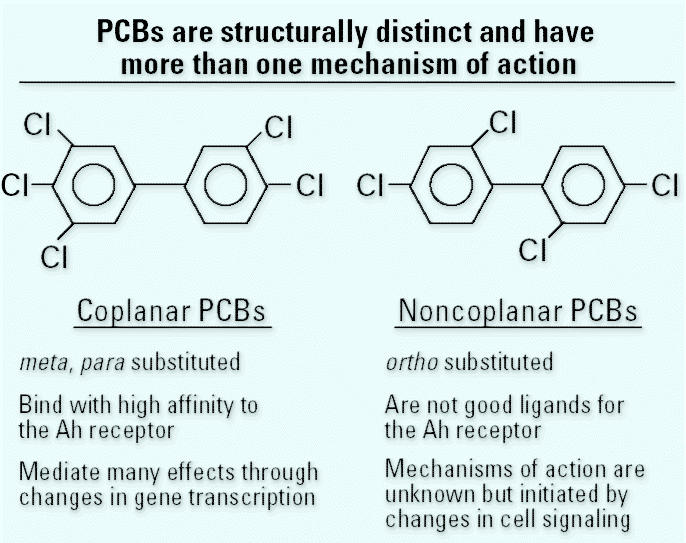
Structure of coplanar and noncoplanar PCBs. 3,3′,4,4′,5-Pentachlorobiphenyl is the representative coplanar PCB depicted. 2,2′,4,4′-Tetrachlorobiphenyl is the representative noncoplanar PCB depicted.

As mentioned above, PCBs comprise 209 individual congeners, and a variety of toxic effects mediated by multiple mechanisms accompany this structural diversity. Effects include neurotoxicity, induction of enzymes involved in xenobiotic metabolism, alterations in reproductive function, hepatotoxicity, carcinogenicity, and effects on cells that mediate innate and specific immunity (Safe 1994). In applying Occam’s Razor, one can think of PCBs as falling into two groups in terms of structure and mechanisms of action (Figure 1). Coplanar PCBs lack *ortho* substitution, bind with high affinity to the Ah receptor, and mediate many of their effects through changes in gene expression initiated by binding to this receptor. Noncoplanar PCBs, which contain chlorine in one or more of the four *ortho* positions, are poor ligands for the Ah receptor. The mechanisms of their biological effects are in many cases unknown but often involve initial changes in cell signaling (Fischer et al. 1998).

Studies were undertaken to compare the biological activity of Aroclor mixtures of PCBs with the activity of products of their reductive dechlorination. The dechlorination process employed resulted in accumulation of congeners substituted in only the *ortho* and *para* positions and containing fewer chlorines than the starting mixtures (Mousa et al. 1996; Quensen et al. 1998). For example, 2,2′,4-trichlorobiphenyl represented 4% (on a molar basis) of the total mixture before dechlorination and 16% of the dechlorinated product. For more detailed description of the congener profile of the remediation products, the reader is referred to Bae et al. (2001), Ganey et al. (2000), and Mousa et al. (1998).

Table 2 is a summary of the results of examination of biological activity. Coplanar, dioxin-like PCBs induce cytochrome P4501A through an Ah receptor–mediated mechanism (Sanderson et al. 1996), and the potency for this effect can be compared with the potency of dioxin (2,3,7,8-tetrachlorodibenzo-*p*-dioxin) to generate a toxic equivalency factor (TEF) for individual congeners (Safe 1993). TEF values can then be used to determine the toxic equivalents (TEQs) for mixtures of chemicals. This approach has been used for risk assessment of dioxin-like compounds, although it is not without limitation (Li and Hansen 1996; Safe 1998). The ability of products of dechlorination of Aroclor mixtures to induce cytochrome P4501A activity, monitored as ethoxyresorufin*-O*-deethylase activity, was examined in the rat liver hepatoma cell line H4IIE. Parent Aroclors 1242 and 1254 were compared with products of their dechlorination by microorganisms collected from two different sites, Silver Lake, Massachusetts, and River Raisin, Michigan. Aroclors were evaluated at concentrations ranging from 0.04 to 2.5 μg/well (250 μL/well), and the dechlorination products were used at molar equivalent concentrations based on biphenyl concentration (biphenyl concentration is unaffected by dechlorination). Both potency and efficacy of induction of the Aroclor mixtures were diminished by dechlorination (Mousa et al. 1998; Quensen et al. 1998). The decrease in potency was dependent on the extent of removal of the coplanar and mono-*ortho-*substituted PCBs, consistent with the known mechanism of this effect. For example, the TEQ for nondechlorinated Aroclor 1242 derived from the assay was 3.1, whereas the TEQ for the dechlorinated mixture was below the limit of detection (0.06). These values were in agreement with TEQs calculated from the known composition of the nondechlorinated and dechlorinated mixtures, 5.7 and < 0.08, respectively.

*In vitro* fertilization is reflective of reproductive capacity. Epidemiologic studies assessing the effects of human exposure to PCBs on fertility and reproduction have yielded various results: some indicate a negative effect of PCBs on fertility, whereas others report no association (Axmon et al. 2001, 2002; Dallinga et al. 2002; Rozati et al. 2002; Yu et al. 2000). In experimental animals dioxin-like chemicals, including some PCBs, cause reproductive toxicity (Birnbaum and Tuomisto 2000; Peterson et al. 1993; Petroff et al. 2001). For example, administration of heavily chlorinated, noncoplanar PCB congeners to male rats decreases several markers of sperm function (Hsu et al. 2003). Exposure of female mice to the coplanar congener 3,3′,4,4′-tetrachlorobiphenyl decreases reproductive capacity (Huang et al. 1998a), and exposure of pregnant mice to Aroclor 1242 or to 3,3′,4,4′-tetrachlorobiphenyl alters fertility in male offspring (Fielden et al. 2001; Huang et al. 1998b). In addition, coplanar PCBs inhibit *in vitro* fertilization of murine eggs (Huang et al. 1998a). Products of dechlorination of Aroclors 1242 and 1254 were compared with the parent Aroclors for the ability to inhibit *in vitro* fertilization of mouse gametes (Mousa et al. 1996, 1998). Aroclor 1254 decreased the percentage of fertilized eggs and increased the percentage of degenerated eggs at 10 ppm and 20 ppm. The products of reductive dechlorination used at equivalent molar concentrations produced less of an adverse effect on fertilization and did not cause gamete degeneration. Similarly, the negative effects of Aroclor 1242 on fertilization were not observed with its product of dechlorination. Based on the observations that coplanar PCBs and heavily chlorinated, noncoplanar PCBs alter reproductive capacity, this result was consistent with the loss of these congeners due to dechlorination.

Environmental exposure to PCBs has been associated with increased risk of cancer in some but not all studies (Demers et al. 2002; Gammon et al. 2002; Kimbrough et al. 2003; Laden et al. 2002; Lucena et al. 2001; Stellman et al. 2000; Woolcott et al. 2001). The transcription factor activator protein-1 (AP-1) is a protein that regulates gene expression and has been implicated in tumorigenesis. Using the rat liver epithelial cell line WB-344, transfected with AP-1–binding DNA and a luciferase reporter gene, the ability of remediation products of Aroclors to induce AP-1 activity was determined. Native Aroclors (2 μg/mL) caused a 2- to 3-fold increase in induction of AP-1 transcription, whereas dechlorinated products (equivalent molar concentration) had no effect on AP-1–mediated transcription (Mousa et al. 1998). Stimulation of AP-1–mediated transcription is attributed to more heavily chlorinated, noncoplanar PCBs; thus, these results are consistent with the loss of heavily chlorinated congeners upon dechlorination.

Exposure to PCBs has been associated with decreased gestation length in several epidemiologic studies (Bercovici et al. 1983; Taylor et al. 1989; Wassermann et al. 1982). Because uterine contractions actuate parturition, the effects of PCBs on contractility of pregnant rat uteri were examined. Aroclor 1242 stimulated contraction of uteri isolated from pregnant rats in a concentration- and time-dependent manner (Bae et al. 1999, 2001). A concentration of 100 μM nondechlorinated Aroclor 1242 increased contraction frequency, whereas smaller concentrations were without effect (Bae et al. 2001). The potency of various Aroclor mixtures to increase uterine contraction frequency was inversely related to chlorine content, suggesting that this effect was mediated by less heavily chlorinated congeners. Results with native Aroclors were compared with the effects of Aroclors that had been dechlorinated by microorganisms collected from the Hudson River basin. Compared with the response to unaltered Aroclor 1242, the dechlorinated mixture shifted the concentration–response curve to the left, such that 10 μM of the dechlorinated mixture caused an increase in uterine contraction frequency. Similarly, the cumulative concentration–response curve of the dechlorinated Aroclor 1254 was shifted to the left relative to that of the unaltered Aroclor 1254. In fact, parent Aroclor 1254 did not stimulate contractions with exposure up to 300 μM, yet the dechlorinated mixture exerted a powerful stimulatory response in terms of both effective concentration range (30 μM increased contraction frequency) and efficacy. Thus, dechlorination produced a mixture with uterine-stimulating activity from a relatively nonactive Aroclor mixture.

PCB exposure has been associated with alterations in immune status in humans (Belles-Isles et al. 2002; Van Den Heuvel et al. 2002) and experimental animals (Arena et al. 2003; De Krey and Kerkvliet 1995; De Krey et al. 1994). In addition, cells of both specific (e.g., lymphocytes) and innate (e.g., neutrophils) immunity are affected by PCBs (Fernlof et al. 1997; Ganey et al. 1993; Suh et al. 2003). For example, noncoplanar PCBs stimulate neutrophils to produce reactive oxygen species, specifically superoxide anion (Ganey et al. 1993). In addition, PCBs increase superoxide anion production in response to subsequent stimulation with phorbol myristate acetate (PMA). The ability of Aroclor 1242 to cause generation of reactive oxygen species in neutrophils was compared with the ability of its products of dechlorination by microorganisms from Silver Lake or River Raisin (Ganey et al. 2000). Exposure of rat neutrophils *in vitro* to Aroclor 1242 at 10 μg/mL increased PMA-stimulated superoxide anion generation. Exposure of neutrophils to products of dechlorination of Aroclor 1242 at equivalent molar concentrations caused similar increases in superoxide anion production (Ganey et al. 2000). Accordingly, dechlorination did not diminish the ability of the mixtures to activate neutrophils. On the other hand, parent Aroclor 1254 did not increase superoxide anion production in PMA-stimulated neutrophils, but its dechlorination products did. Thus, like the effects observed for stimulation of uterine contractility, dechlorination induced biological activity in a nonactive Aroclor mixture. These results are consistent with the accumulation of noncoplanar PCBs in the dechlorination products.

Increased incidence of diabetes has been associated with high concentrations of PCBs or other organochlorine chemicals in serum (Glynn et al. 2003; Longnecker et al. 2001). In addition, Aroclor mixtures of PCBs stimulate the release of insulin from the rat clonal cell line RINm5F (Fischer et al. 1996). This effect is mediated by noncoplanar PCBs (Fischer et al. 1998). RINm5F cells were exposed to Aroclor 1242 or 1254 (10 μg/mL) or their products of dechlorination by River Raisin or Silver Lake microorganisms (equivalent molar concentrations), and insulin release was examined. Both parent Aroclor mixtures caused release of insulin within 30 min of exposure. The magnitude of response to the mixtures of dechlorinated Aroclors was similar or greater when compared with the non-dechlorinated parent mixtures (Ganey et al. 2000). These results are consistent with the observed accumulation of *ortho*-substituted, noncoplanar PCBs in the mixtures produced by reductive dechlorination.

Taken together, these results demonstrate that a variety of responses can be observed after exposure of mammalian cell systems to products of remediation. In the case of the studies described above for remediation of PCBs, the responses followed what would be expected based on structure and known biological activity of the chemicals. That is, Ah receptor–mediated activities diminished because of the removal of coplanar congeners via *meta* and *para* dechlorination processes, and biological activities mediated by non-coplanar PCBs were enhanced or unchanged. These studies were guided by knowledge of some of the mechanisms of action of PCBs. For remediation processes aimed at chemicals for which less is known about effects in mammalian systems, studies similar to those described above may reveal unexpected results.

## Summary and Future Needs

Several important aspects of evaluation were not addressed in this series of experiments. For this specific case of remediation of PCBs, no measure of neurotoxicity was performed. This is an important deficit because the neurotoxic effects of PCBs have been demonstrated experimentally and suggested by results of epidemiologic studies (Schantz et al. 2001, 2003; Seegal 1996). Because many neurotoxic effects are associated with non-coplanar PCBs (Kodavanti and Tilson 1997; Wong et al. 2001), one would expect effects of the products of remediation to be similar or greater than those of the parent Aroclors.

All the assays used were *in vitro* assays that represent selected functions that occur within a whole organism. This approach does not address issues of exposure, including relevant routes of exposure to environmental contaminants and their remediation products. In addition, the duration of exposure during *in vitro* assays is short and does not mimic longer-term, often-repeated exposures that occur naturally. Issues of bioavailability are not considered when performing *in vitro* assays. This includes bioavailability from an environmental engineering point of view (e.g., how much of the contaminant is not bound to soil constituents) and from the perspective of toxicology (e.g., how much of the exposure dose interacts with target tissue). These issues can best be addressed using whole-organism studies, which, as mentioned above, are costly and inconvenient. In addition, biologically based toxicokinetic and toxicodynamic modeling could be used to address issues of extrapolation to human risk. In the future, approaches to include these considerations must be developed.

Thus, it should be emphasized that the approach described above to evaluate effects of products of remediation in a variety of *in vitro* assays employing mammalian cells represents a beginning. Using this approach, the biological activity of remediation products is compared with activity of the parent compound, such that relative activity is assessed. Although this is a useful component in determination of the effectiveness of remediation, it stops short of estimating potential health risk of the remediation products. Comprehensive evaluation of the biological activity of remediation products will necessitate far more extensive *in vitro* and *in vivo* testing, the use of validated extrapolation models to assess risk to people and wildlife, and epidemiologic correlates. It seems unlikely that this type of effort will arise from any single institution. It is more likely to be achieved through a consortium of institutions or a government-based testing facility that can amass the expertise and resources required.

Despite these limitations, several points can be drawn from these remediation assessment evaluations. First, the overarching message is that it is important to evaluate the biological activity of products of remediation and also of stable intermediates produced during remediation. As seen in the series of experiments presented above, the products of remediation are not necessarily devoid of biological activity. When compared with the parent compound, activity of remediation products may be decreased, unchanged, or increased. It is also possible that biological activity of remediation products may be qualitatively different from the activity of the starting compound. Furthermore, although not observed in the studies described above, when bacteria are used in remediation processes, it is possible that bacterial by-products unrelated to the chemical contaminant itself are produced that have biological activity in some cellular systems. Another important point to be made is that a better understanding of the mechanisms of biological effects of contaminants will permit a more directed approach to evaluation of the activity of the remediation products. The selection of bioassays to be used as well as the specific details of experimental design can be based on known mechanisms of action of the parent compounds. Finally, knowledge of the spectrum of biological activities associated with remediated chemicals and their stable intermediates will provide the basis for more accurate risk assessment and guide remediation needs and approaches.

## Figures and Tables

**Table 1:** Examples of assays used to assess the biological activity of remediation products.

Assay	Biological functions represented
Induction of cytochrome P450 enzymes	Receptor-mediated activity (Ah receptor)
Activation of mitogen-activated protein kinases	Intracellular signaling
Disruption of gap junctional intercellular communication	Intercellular signaling, cell death
Activation of AP-1 transcription factor	Gene expression
Alteration in neutrophil function	Cellular function, cell death
Stimulation of insulin release	Cellular function
Contraction of uterine muscle *in vitro*	Integrated tissue function
Alteration in fertilization *in vitro*	Integrated tissue/organ system function
Stimulation of lymphocyte proliferation	Proliferation, cell death

**Table 2:** Summary of effects of biological activity of dechlorinated PCBs.

Biological activity	Effect of parent Aroclor	Type of PCBs mediating effect	Effect of dechlorinated products	Reference
Cytochrome P450 activity	Induction	Coplanar	None	Mousa et al. 1998; Quensen et al. 1998
*In vitro* fertilization	Reduction	Coplanar	None	Mousa et al. 1996, 1998
AP-1-mediated transcription	Induction	More heavily chlorinated, noncoplanar	None	Mousa et al. 1998
Uterine contraction	Stimulation	Less heavily chlorinated, noncoplanar	Greater stimulation	Bae et al. 2001
Neutrophil function	Activation	Noncoplanar	Same or greater activation	Ganey et al. 2000
Insulin secretion	Stimulation	Noncoplanar	Stimulation	Ganey et al. 2000
